# Transition of D3c branch and novel recombination events contribute to the diversity of Coxsackievirus A6 in Beijing, China, from 2019 to 2023

**DOI:** 10.1093/ve/veaf036

**Published:** 2025-05-11

**Authors:** Xuejie Zhang, Renqing Li, Roujian Lu, Changcheng Wu, Zhichao Liang, Zhongxian Zhang, Baoying Huang, Yang Yang, Zhenyong Qi, Daitao Zhang, Desheng Zhai, Quanyi Wang, Wenjie Tan

**Affiliations:** School of Public Health, Xinxiang Medical University, No. 601 Jinsui Avenue, Hongqi District, Xinxiang 453003, Henan, China; Institute for Infectious Disease and Endemic Disease Control, Beijing Center for Disease Prevention and Control, No. 16 Hepingli Middle Road, Dongcheng District, Beijing 100013, China; National Key Laboratory of Intelligent Tracking and Forecasting for Infectious Diseases, NHC Key Laboratory of Biosafety, National Institute for Viral Disease Control and Prevention, Chinese Center for Disease Control and Prevention, No. 155 Changbai Road, Changping District, Beijing 102206, China; National Key Laboratory of Intelligent Tracking and Forecasting for Infectious Diseases, NHC Key Laboratory of Biosafety, National Institute for Viral Disease Control and Prevention, Chinese Center for Disease Control and Prevention, No. 155 Changbai Road, Changping District, Beijing 102206, China; Institute for Infectious Disease and Endemic Disease Control, Beijing Center for Disease Prevention and Control, No. 16 Hepingli Middle Road, Dongcheng District, Beijing 100013, China; National Key Laboratory of Intelligent Tracking and Forecasting for Infectious Diseases, NHC Key Laboratory of Biosafety, National Institute for Viral Disease Control and Prevention, Chinese Center for Disease Control and Prevention, No. 155 Changbai Road, Changping District, Beijing 102206, China; National Key Laboratory of Intelligent Tracking and Forecasting for Infectious Diseases, NHC Key Laboratory of Biosafety, National Institute for Viral Disease Control and Prevention, Chinese Center for Disease Control and Prevention, No. 155 Changbai Road, Changping District, Beijing 102206, China; Institute for Infectious Disease and Endemic Disease Control, Beijing Center for Disease Prevention and Control, No. 16 Hepingli Middle Road, Dongcheng District, Beijing 100013, China; School of Public Health, Xinxiang Medical University, No. 601 Jinsui Avenue, Hongqi District, Xinxiang 453003, Henan, China; Institute for Infectious Disease and Endemic Disease Control, Beijing Center for Disease Prevention and Control, No. 16 Hepingli Middle Road, Dongcheng District, Beijing 100013, China; School of Public Health, Xinxiang Medical University, No. 601 Jinsui Avenue, Hongqi District, Xinxiang 453003, Henan, China; Institute for Infectious Disease and Endemic Disease Control, Beijing Center for Disease Prevention and Control, No. 16 Hepingli Middle Road, Dongcheng District, Beijing 100013, China; School of Public Health, Xinxiang Medical University, No. 601 Jinsui Avenue, Hongqi District, Xinxiang 453003, Henan, China; National Key Laboratory of Intelligent Tracking and Forecasting for Infectious Diseases, NHC Key Laboratory of Biosafety, National Institute for Viral Disease Control and Prevention, Chinese Center for Disease Control and Prevention, No. 155 Changbai Road, Changping District, Beijing 102206, China

**Keywords:** Coxsackievirus A6 (CVA6), hand, foot, and mouth disease (HFMD), novel D3c branch, recombinant forms (RFs), metagenomic next-generation sequencing

## Abstract

Coxsackievirus A6 (CVA6) is a major pathogen responsible for numerous outbreaks of hand, foot, and mouth disease (HFMD) worldwide. This study investigates the molecular evolution and recombination of CVA6 in Beijing, China. Full-length sequences of 54 CVA6 from Beijing (2019–2023) were obtained through metagenomic next-generation sequencing and Sanger sequencing. These sequences were compared with representative sequences from GenBank to analyse their phylogenetic characteristics, recombination diversity, and evolutionary dynamics. The 54 CVA6 strains co-circulated with those from multiple provinces in China, as well as from South Korea and Japan. Phylogenetic analysis revealed a novel D3c branch, with the VP1 T283A amino acid mutation identified as a key change in its formation. One sequence belonged to the D3a branch, while 53 sequences belonged to the D3c branch. Recombination analysis identified RF-A (46, 85.1%) and three novel recombinant forms (RFs): RF-Z (1, 1.9%), RF-AA (1, 1.9%), and RF-AB (6, 11.1%). Bayesian phylogenetic analysis estimated that the most recent common ancestor of D3c emerged in August 2013 (95% highest probability density (HPD): May 2012 to September 2014), with recombination events occurring in RF-Z (2017–2019), RF-AA (2019–2023), and RF-AB (2021–2023). In conclusion, we revealed a globally circulating CVA6 D3c branch and identified three novel RFs, providing valuable insights for the intervention and control of HFMD.

## Introduction

Hand, foot, and mouth disease (HFMD) is a self-limiting viral infection that primarily affects children under 5 years of age. It is characterized by symptoms such as fever, maculopapular rashes, and vesicular lesions on the hands, feet, and oral mucosa (Faridi et al. 2024). While HFMD is typically mild, severe cases can lead to complications including meningitis, myocarditis, and neurological symptoms, which can result in fatal outcomes or long-term sequelae (Li et al. 2021). Since the first report of HFMD in 1957, Enterovirus A71 (EV-A71) and Coxsackievirus A16 (CV-A16) have been identified as the primary causative agents of epidemic outbreaks worldwide. In 2008, Coxsackievirus A6 (CVA6) was first recognized as a pathogen associated with HFMD outbreaks in Finland (Osterback et al. 2009), and has since been responsible for widespread epidemics across Europe (Mirand et al. 2012, Martínez-López et al. 2021), the Americas (Cisterna et al. 2019, Luchs et al. 2022), and Asia (Puenpa et al. 2022, Foronda et al. 2023). In mainland China, the number of CVA6-associated HFMD cases has notably increased since 2013 (Liu et al. 2021). Introduction of the EV-A71 vaccine in 2016 led to a decline in EV-A71-related HFMD cases (Xiao et al. 2022), making CVA6 the predominant pathogen in several regions and presenting new challenges for epidemic control.

The CVA6 virus is spherical with icosahedral symmetry, lacks an envelope or protrusions, and has an approximate diameter of 30 nm. Its genome consists of a non-segmented, single-stranded, positive-sense RNA, ~7400 nt in length (Song et al. 2017). Upon entering host cells, the CVA6 RNA directly functions as an mRNA and is translated into a large polyprotein of ~2200 amino acids. This polyprotein is subsequently cleaved by viral proteases into P1, P2, and P3 polyproteins (Ypma-Wong and Semler 1987). The P1 region encodes the capsid proteins (VP1–VP4), whereas the P2 and P3 regions encode the non-structural proteins (2A–2C, 3A–3D) (Jiang et al. 2014). VP1 is the primary serotype-specific protein that is widely used in viral identification and evolutionary studies (Oberste et al. 1999).

Song et al. (2017) classified global CVA6 sequences collected prior to 2015 into four genotypes, namely A, B (B1–B2), C (C1–C2), and D (D1–D3), using a nucleotide sequence divergence threshold of 15% within the VP1 region. The sub-genotype D3 was further subdivided into D3a and D3b, with a mean genetic distance of 6% between these two branches. Lu et al. (2024) classified CVA6 into six genotypes (A–F). Recently, all CVA6 sequences have been classified as sub-genotype D3, with a notable increase in genetic divergence compared to earlier sequences. Puenpa et al. (2022) further subdivided sub-genotype D3 into subclades D3.1–D3.7. Therefore, it is necessary to apply bioinformatic methods to explore the genetic evolution of CVA6, enhance the understanding of its genetic characteristics, and elucidate its transmission patterns.

Recombination is a common phenomenon observed in enteroviruses (Ma et al. 2023, Shi et al. 2023, Yang et al. 2023b). The error-prone 3Dpol RNA-dependent RNA polymerase (RdRp) of enteroviruses leads nucleotide misincorporations during genome replication. In turn, recombination can help prevent the accumulation of deleterious mutations, which may explain the observed high-frequency recombination events (Wang and Wen 2024). With this rapid increase in prevalence, global CVA6 variants have been classified into 25 recombinant forms (RFs), labelled RF-A to RF-Y (Puenpa et al. 2022, Lu et al. 2024). The conservation of the CVA6 capsid gene resulted in high transmissibility, but the lineage-specific non-capsid gene may influence pathogenicity (Song et al. 2020). Therefore, enhanced surveillance and whole-genome sequencing of CVA6 are essential for detecting new recombination events and improving public health responses.

In this study, we aimed to compare 54 newly sequenced CVA6 genomes with globally reported CVA6 VP1 sequences to assess their phylogenetic relationships. Additionally, the rationale was to conduct recombination analyses to identify emerging recombinant variants and associated amino acid mutations, providing valuable insights into the genetic evolution and molecular epidemiology of CVA6.

## Materials and methods

### Clinical sample collection

Clinical samples were collected from patients diagnosed with HFMD at the outpatient and inpatient departments of hospitals in Beijing between May 2019 and August 2023. All samples were confirmed to be positive for CVA6 by real-time quantitative PCR (RT-qPCR) and were screened to exclude other enterovirus infections. A total of 5 vesicular fluid samples and 49 throat swab samples positive for CVA6 were obtained. After collection, all samples were immediately stored in a dedicated virus preservation solution and stored at −80°C for subsequent analysis. This study was approved by the Ethics Committee of Beijing Center for Disease Prevention and Control (Approval No. BJCDC/GD12-KJ-F03). Written informed consent was obtained from the legal guardians of all paediatric patients or directly from the adult patients themselves.

### Sample processing and nucleic acid amplification

Samples were processed to remove large particle contaminants by high-speed centrifugation, followed by viral nucleic acid extraction and reverse transcription. To achieve non-selective amplification of viral nucleic acids, sequence-independent single-primer amplification (SISPA) was employed (Chrzastek et al. 2017). The process included the following steps. (i) Centrifugal purification: a 200 μl sample was vortexed with 200 μl of PBS for 5 min and then centrifuged at 13 000 rpm for 10 min to eliminate large particles. The supernatant was then filtered through a 0.45 μm filter to remove smaller impurities, followed by treatment with Benzonase Nuclease, TURBO™ DNase, and RNase I to degrade free nucleic acids. (ii) Viral nucleic acid extraction: performed with the QIAamp MinElute Virus Spin Kit (Qiagen). (iii) Reverse transcription: conducted with the SuperScript III First-Strand Synthesis System for RT-PCR (ShiningSun). (iv) Double-stranded DNA synthesis: performed with the Klenow Fragment (NEB). (v) SISPA amplification: performed with AccuPrime™ Taq DNA Polymerase (Thermo Fisher). The amplified products were purified with the QIAquick PCR Purification Kit (Qiagen). For targeted amplification, CVA6-specific primers were designed for specific samples (Supplementary Table S1).

### Library preparation and metagenomic next-generation sequencing

Libraries were constructed using the Nextera XT DNA Sample Preparation Kit (Illumina) and purified with AMPure XP Beads (Beckman). Library fragments were verified using an Agilent 2100 Bioanalyzer (Agilent). Following quality control, sequencing was performed on an Illumina NovaSeq 6000 platform.

### Metagenomic next-generation sequencing data analysis

Fastp (version 0.23.2) (Chen et al. 2018b) was used to filter low-quality reads from the raw sequencing data. BWA (version 0.7.17) (Li and Durbin 2009) was employed to map clean reads to the human reference genome (hg38) and remove the host reads. Microorganisms annotation was performed using Kraken2 (Wood et al. 2019) in combination with Bracken (Lu et al. 2017). For the CVA6 sequences, assembly was performed using Minimap2 (version 2.24) (Li 2018) and SPAdes (version 3.13.0) (Prjibelski et al. 2020). For sequences generated by next-generation sequencing, Sanger sequencing was used to fill the gaps and verify the assembled sequences.

### Dataset construction

A total of 4972 CVA6 VP1 sequences were retrieved from GenBank (as of 31 October 2024). After excluding low-quality sequences, 4947 sequences were retained. Clustering analysis was performed using the USEARCH tool with a 96% nucleotide identity threshold, resulting in the selection of 122 representative sequences. The 54 newly sequenced CVA6 VP1 sequences were then combined with the representative ones. TempEst (version 1.5.3) was used to assess the correlation between nucleotide sequence divergence and sampling time (Rambaut et al. 2016). Four sequences with significant temporal bias were excluded, resulting in Sequence Library 1, which contains 172 VP1 sequences (Supplementary Table S2). Furthermore, maximum likelihood (ML) trees were constructed separately for global and Beijing sequences (Supplementary Figs S1 and S2). The 54 sequences were combined with 4682 global D3 sub-genotype sequences to form Sequence Library 2 (Supplementary Table S3), and with 307 Beijing D3 sub-genotype sequences to form Sequence Library 3 (Supplementary Table S4). Additional sequences from GenBank (as of 15 February 2025) were included to identify parental sequences for the RF-AA analysis. For further details, please refer to the Supplementary material.

### Phylogenetic analysis

Sequences were aligned using MAFFT (version 7.487) (Katoh and Standley 2013), and a ML tree was constructed using IQ-TREE (version 2.3.6) (Nguyen et al. 2015) with the general time-reversible (GTR) nucleotide substitution model. Statistical support for the tree nodes was assessed using a bootstrap procedure with 1000 replicates. ML trees were generated and visualized using Interactive Tree of Life (iTOL; https://itol.embl.de/). Sequence similarity was calculated using the BioEdit (version 7.7.1.0) (Hall 1999).

### Recombination analysis

To detect recombination events among the 54 CVA6 sequences in this study, a ML tree was constructed based on the 3Dpol region, using representative sequences from GenBank along with the 54 CVA6 sequences. After retrieving the 2A, 2B, 2C, 3AB, 3C, and 3D regions from the eight recombinant CVA6 sequences, a BLAST search was performed to obtain full sequences with over 90% similarity from GenBank as reference sequences for analysis. Simplot (version 3.5.1) (Salminen et al. 1995) was used to estimate recombination signals, generating similarity plots and bootscanning analyses to identify potential recombination events. The final step involved using seven methods in the Recombination Detection Program 4 (RDP4, version 4.101) (Martin et al. 2015), including RDP, GENECONV, Chimera, MaxChi, BootScan, SiScan, and 3Seq, to explore breakpoints and identify the major and minor parental strains.

### Amino acid site variation analysis

The VP1 amino acid sequences of 4736 CVA6 sequences from Sequence Library 2 were analysed using BioEdit, and the site was considered highly variable when the amino acid entropy exceeded 0.6. The proportion of highly variable residues in the VP1 amino acid sequences was visualized using the WebLogo online tool (https://weblogo.threeplusone.com/create.cgi). The atomic model of the CVA6 capsid particle (PDB ID: 5YHQ) was obtained from the Protein Data Bank (PDB, https://www.rcsb.org/) and was visualized in PyMOL (version 2.6) (Rigsby and Parker 2016, Chen et al. 2018a). Synonymous sites were identified using MEGA software (version 11.0.13) based on codon analysis (Tamura et al. 2021).

### Temporal dynamics analysis

Bayesian phylogenetic analysis was performed using BEAST (version 1.10.4) (Bouckaert et al. 2014). Phylogenies were inferred using the GTR + G + I model, a relaxed molecular clock with an uncorrelated lognormal distribution, and a Bayesian skyline coalescent model (Drummond et al. 2005). Calculations were performed with chain lengths of 90 000 000 to ensure Markov Chain Monte Carlo (MCMC) convergence, and the results were imported into Tracer (version 1.7.2) (Rambaut et al. 2018) for visualization. Convergence was considered to have been achieved when the effective sample size exceeded 200. The Bayesian maximum clade credibility (MCC) tree was constructed using TreeAnnotator (version 1.10.4), with the burn-in option set to discard the first 10% of data from the sample tree.

### Statistical analysis

All analyses were conducted using IBM SPSS Statistics 20. The number of amino acid mutations was compared between recombinant and non-recombinant regions using the Chi-square (*χ*^2^) test. *P* < .05 was defined as significant.

## Results

### The homologous analysis of 54 Coxsackievirus A6 sequences

Fifty-four full-length CVA6 sequences were amplified and sequenced, including 12 from 2019, 18 from 2020, 1 from 2021, 5 from 2022, and 18 from 2023. The VP1 region is 915 nt, encoding 305 amino acids. Nucleotide and amino acid sequence identity analyses were performed on 54 CVA6 VP1 sequences from this study and the CVA6 prototype strain (Gdula, USA/1949). The nucleotide and amino acid sequence identities of the 54 CVA6 VP1 sequences ranged from 92.0% to 100.0% and 97.0% to 100.0%, respectively. Compared to the prototype strain (Gdula), the nucleotide and amino acid sequence identities were 81.9%–84.1% and 94.7%–96.0%, respectively.

The ORF region of the 54 CVA6 sequences in this study showed the highest nucleotide identity (96.05%–99.74%) with sequences collected from China (including Beijing, Henan, Jilin, Yunnan, Chongqing, and Heilongjiang), South Korea, and Japan between 2017 and 2023. All of these sequences were classified as sub-genotype D3 (Supplementary Table S5). These results suggest the co-evolution and co-circulation of CVA6 in Beijing and other regions of China, as well as South Korea and Japan, between 2019 and 2023.

### D3c branch is the predominant evolutionary branch

**Figure 1:**
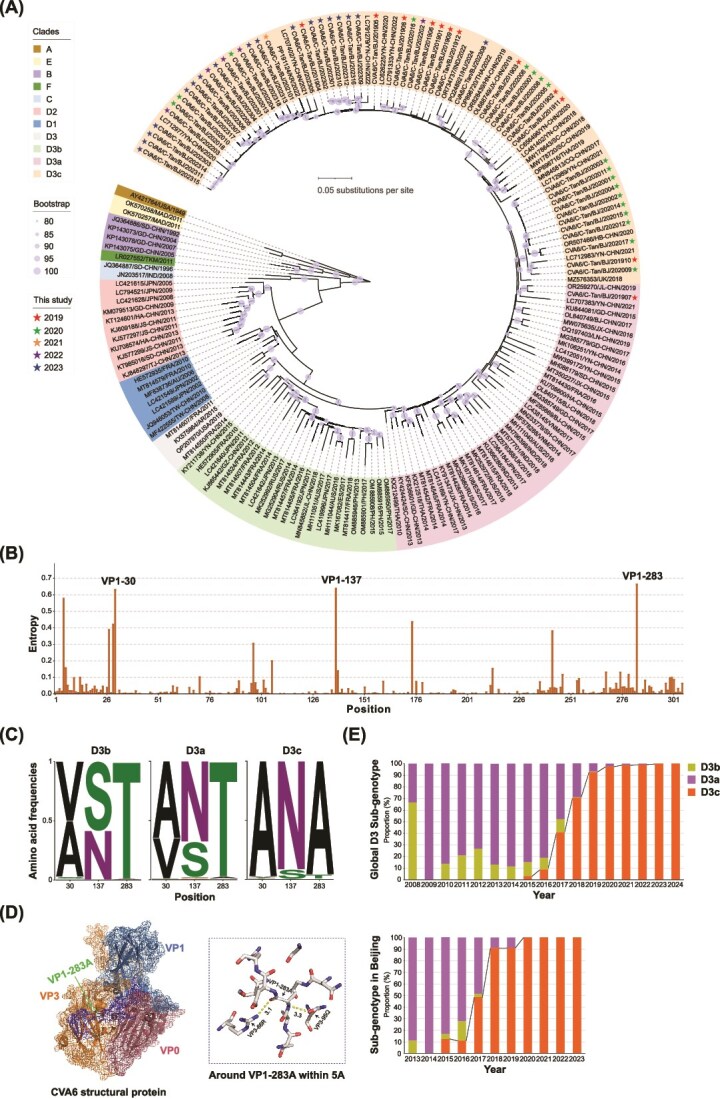
The D3c branch is the predominant evolutionary branch. (A) Phylogenetic analysis of VP1 sequences (nucleotide positions 2441–3355, 915 nt, relative to AY421764) using 172 CVA6 sequences from Sequence Library 1. ML tree was constructed with 1000 bootstrap replicates to assess the robustness of the groupings, with bootstrap values ≥80% indicated. (B) Entropy values at each position of the 4736 VP1 amino acid sequences from Sequence Library 2. (C) Composition of VP1 residues at positions 30, 137, and 283 in the D3a, D3b, and D3c branches. (D) Atomic model of the CVA6 particle capsid, obtained from the Protein Data Bank (PDB ID: 5YHQ), visualized using PyMOL (version 2.6). VP0 is shown in raspberry, VP1 in sky blue, VP3 in tangerine orange, and VP1-283A in green. The C-terminus of VP1 is labelled in blue. VP1-283A forms hydrogen bonds with VP3-95Q and VP3-66R. (E) Yearly distribution of the CVA6 D3 sub-genotype (D3a, D3b, and D3c from Sequence Library 2) worldwide from 2008 to 2024 (top). Yearly distribution of the CVA6 D3 sub-genotype (D3a, D3b, and D3c from Sequence Library 3) in Beijing from 2013 to 2023 (bottom).

A total of 172 CVA6 sequences from Sequence Library 1 were subjected to phylogenetic analysis based on complete VP1 sequences. The cladogram indicated that all CVA6 sequences clustered into six genotypes, designated as A, B, C, D, E, and F. Genotype D was further subdivided into sub-genotypes D1–D3. Among these, sub-genotype D3 was further divided into three evolutionary branches: D3a, D3b, and D3c. The newly generated 53 sequences from 2019 to 2023 were classified into the D3c branch, and one sequence from 2019 was classified into the D3a branch (Fig. 1A).

Additionally, we aligned 4736 full VP1 sequences from Sequence Library 2 at each amino acid site. Entropy analysis showed that VP1-30, VP1-137, and VP1-283 are highly variable amino acid sites (Fig. 1B). Further analysis of the amino acid composition at these sites within the D3 sub-genotype sequences showed significant differences. At VP1-283, notable variations were observed across different branches. In the D3b branch, threonine (Thr) accounted for 98.9%, with alanine (Ala) constituting 1.1%. In the D3a branch, Thr comprised 97.5%, Ala 1.8%, and other amino acids 0.7%. In contrast, in the D3c branch, Ala, Thr, and other amino acids accounted for 95.9%, 3.5%, and 0.6%, respectively (Fig. 1C). The VP1-283 site is involved in the formation of CVA6 conformational epitopes (Liping et al. 2020), and evolutionary analysis indicated a shift over time from the hydrophilic Thr to the hydrophobic Ala at this position (Supplementary Fig. S3). Structural modelling of the CVA6 capsid suggested that when VP1-283 is Ala, it forms hydrogen bonds with VP3-66 (arginine, Arg) and VP3-95 (glutamine, Gln) (Fig. 1D).

We analysed the global prevalence of the D3 sub-genotype. The D3b and D3a branches were first identified globally in 2008, with D3b being more prevalent. Between 2009 and 2016, CVA6 outbreaks were predominantly driven by the D3a branch, although D3b strains appeared sporadically during this period. Since 2015, the number of D3a strains has gradually decreased, while the number of D3c strains has significantly increased (Fig. 1E and Supplementary Table S3). We also analysed the D3 sub-genotype in Beijing. The D3a and D3b branches were first identified in Beijing in 2013. From 2013 to 2016, CVA6 outbreaks in Beijing were primarily driven by the D3a strains, with D3b strains sporadically detected in 2013, 2015, 2016, and 2017, comprising a small proportion. The D3c branch was first detected in Beijing in 2015, and its proportion has gradually become dominant from 2016 to 2023 (Fig. 1E and Supplementary Table S4). These data suggest that D3c strains have gradually become the predominant cause of CVA6 outbreaks in recent years.

Based on the limited early genomic sequencing data, spatiotemporal analysis of 1521 CVA6 D3c branch sequences (Supplementary Table S6) revealed that the D3c strains initially spread within China during 2015 and 2016. Further analysis indicated that D3c strains have been detected in 11 countries, including those in Asia (1448 from China, 7 from Japan, 16 from South Korea, 8 from India, 20 from Thailand, and 6 from Vietnam), Europe (4 from France, 3 from Hungary, 2 from Russia, and 6 from the UK), and Oceania (1 from Australia).

### Three novel recombination forms in eight Coxsackievirus A6 sequences

**Figure 2:**
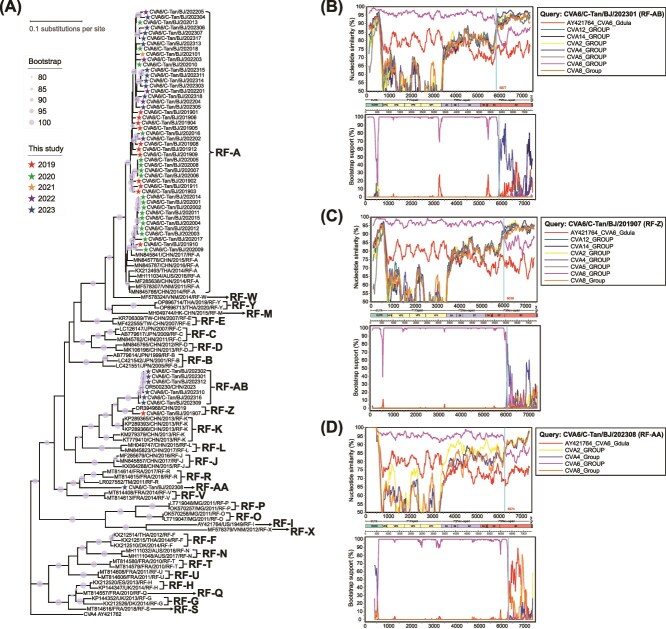
Three novel recombination forms in eight CVA6 sequences. (A) Phylogenetic comparison of partial 3Dpol sequences (nucleotide positions 6073–6884, 812 nt, relative to AY421764) using 54 sequences and representative CVA6 sequences obtained from GenBank. ML tree was constructed with 1000 bootstrap replicates to assess the robustness of the groupings, with bootstrap values ≥80% shown. Recombination events from (B) CVA6/C-Tan/BJ/202301, (C) CVA6/C-Tan/BJ/201907, and (D) CVA6/C-Tan/BJ/202308 sequences were identified through similarity plots and bootscanning analyses, using a sliding window of 200 nt with 20 nt steps.

To investigate the novel RFs of the 54 CVA6 sequences, we classified them based on the 3Dpol region into distinct RFs. Phylogenetic analysis revealed that the 3Dpol nucleotide sequences clustered into 28 well-supported lineages, ranging from RF-A to RF-AB (Fig. 2A). The majority of new sequences (85.1%, 46/54) were assigned to the RF-A lineage. Notably, the sequence from CVA6/C-Tan/BJ/201907 (1.9%, 1/54) clustered with a sequence from Jilin Province (2019, GenBank: OR394968), forming the RF-Z lineage. The CVA6/C-Tan/BJ/202308 (1.9%, 1/54) formed an independent branch, which we designated the RF-AA lineage. Additionally, six sequences from 2023 (CVA6/C-Tan/BJ/202301, CVA6/C-Tan/BJ/202302, CVA6/C-Tan/BJ/202309, CVA6/C-Tan/BJ/202310, CVA6/C-Tan/BJ/202312, and CVA6/C-Tan/BJ/202316) (11.1%, 6/54) clustered with a sequence from Henan Province (2023, GenBank: OR500230) to form the RF-AB lineage.

The results of SimPlot and BootScan identified recombination signals in the three newly identified RFs. The recombination pattern of the RF-AB sequences was consistent, as demonstrated by CVA6/C-Tan/BJ/202301 (Fig. 2B), with additional related results provided in Supplementary Fig. S4. In the P1, P2, and 5′ half of the P3 regions, RF-Z, RF-AA, and RF-AB sequences exhibited high similarity to the CVA6 group. However, in the 3′ end of the 3C region, the 3D region, and the 3′ untranslated region (UTR), the RF-AB sequences displayed higher similarity to the CVA4 group (Fig. 2B), whereas the RF-Z sequence showed greater similarity to both the CVA2 and CVA4 groups (Fig. 2C). In the 3D region and the 3′ UTR, the RF-AA sequence exhibited higher similarity to the CVA2, CVA4, and CVA8 groups (Fig. 2D).

**Figure 3:**
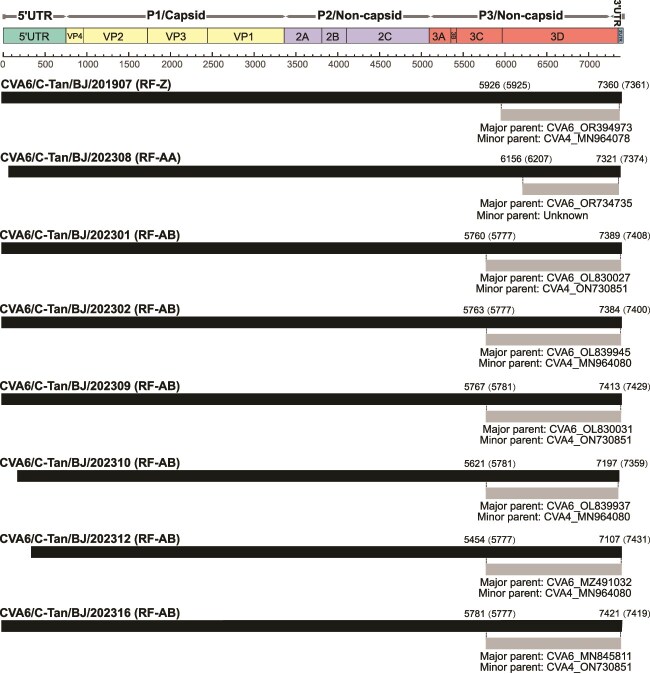
Genomic map of recombination events in eight CVA6 sequences predicted by RDP4. The black band represents the full-length genome of the CVA6, with numbers above indicating the start and end positions of the breakpoints (numbers in brackets represent the breakpoint positions aligned with AY421764). The grey bands represent the recombined genomic regions, with the text below indicating the major and minor parents of the predicted recombination event.

The results of RDP4 provided detailed breakpoints for the three novel RFs (Fig. 3 and Supplementary Table S7). The breakpoint positions of the RF-Z were located between 5926 and 7360 nt, covering the 3′ end of the 3C region, the 3D region, and the 3′ UTR. The major and minor parent strains were identified as CVA6 (GenBank: OR394973) and CVA4 (GenBank: MN964078), respectively, as supported by seven methods. For the RF-AB, the breakpoint positions were primarily located at 5777 and 7431 nt (aligned with AY421764), covering the 3′ end of the 3C region, the 3D region, and the 3′ UTR. The major and minor parent strains were identified as CVA6 (GenBank: OL830027, OL839945, OL830031, OL839937, MZ491032, and MN845811) and CVA4 (GenBank: ON730851 and MN964080), respectively, also supported by seven methods. The breakpoint positions of the RF-AA sequence were located between 6156 and 7321 nt, encompassing the 3D region and the 3′ UTR. Notably, CVA6 (GenBank: OR734735) was identified as the major parent, while the minor parent within the 6156–7321 nt region remained unclear, as supported by six distinct methods.

### Parental strains identification for RF-AA in the 6156–7321 nt region

**Figure 4:**
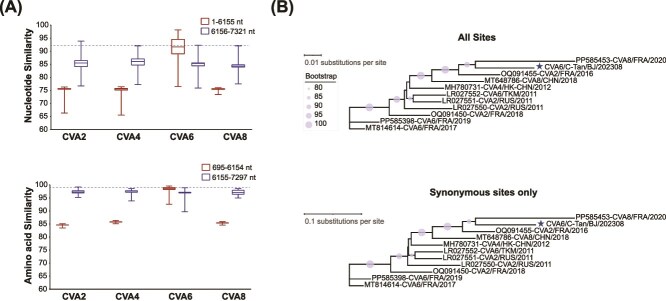
Parental strains identification for RF-AA in the 6156–7321 nt region. (A) Analysis of the nucleotide and amino acid sequence similarity of CVA6/C-Tan/BJ/202308 compared to the complete genomic sequences of CVA2, CVA4, CVA6, and CVA8 available in GenBank. (B) Phylogenetic trees of RF-A and the 10 most similar sequences, estimated from the 6155–7297 nt region (top) and synonymous sites only (bottom). Clade support values obtained from 1000 bootstrap replicates are shown.

The results from SimPlot and BootScan revealed that the RF-AA sequence exhibited higher similarity in the 3D region and the 3′ UTR with the CVA2, CVA4, and CVA8 groups. However, RDP4 did not clearly identify the minor parent for the 6156–7321 nt region. Further analysis of the evolutionary characteristics of the RF-AA sequence indicated that in the 1–6155 nt region, RF-AA displayed the highest nucleotide similarity with the CVA6 group, with a maximum identity of 98.2%. In contrast, the nucleotide similarity between RF-AA and the CVA2, CVA4, CVA6, and CVA8 groups in the 6156–7321 nt region was comparatively low, with maximum values of 93.9%, 92.1%, 92.3%, and 92.1%, respectively. Amino acid similarity analysis between RF-AA and the four groups within the 695–6154 nt and 6155–7297 nt regions revealed that, in the 695–6154 nt region, RF-AA displayed the highest amino acid similarity to CVA6, with a maximum similarity of 99.6%. In the 6155–7297 nt region, RF-AA showed relatively high amino acid similarity to the CVA2, CVA4, CVA6, and CVA8 groups, with maximum similarity values of 99.2%, 98.6%, 98.9%, and 98.6%, respectively (Fig. 4A).

In the 6155–7297 nt region, the nucleotide similarity between the RF-AA sequence and the 10 most similar sequences ranged from 91.5% to 93.8%, while the amino acid similarity varied from 97.6% to 98.6%. Following the methodology for studying the receptor-binding domain (RBD) in SARS-CoV-2 (Lam et al. 2020), we conducted phylogenetic analysis based on both all sites and synonymous sites only within the 6155–7297 nt region. The phylogenetic trees generated from both datasets exhibited highly consistent topologies with the RF-AA clustering with PP585453-CVA8/FRA/2020 (Fig. 4B). Therefore, the observed amino acid similarity between RF-AA and the 10 selected sequences in the 6155–7297 nt region is unlikely to be attributable to selectively mediated convergent evolution. However, conclusively differentiating between recombination and selectively mediated convergent evolution using the current dataset remains challenging.

### Analysis of mutation profiles in the RF-Z, RF-AA, and RF-AB sequences

**Figure 5:**
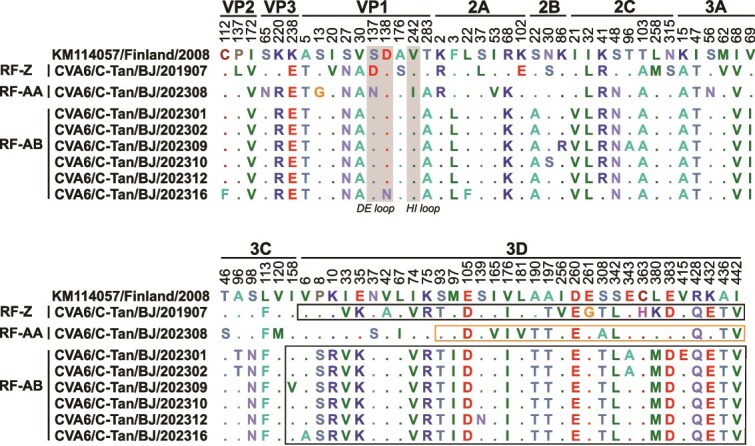
Analysis of mutation profiles in the RF-Z, RF-AA, and RF-AB sequences. The numbers above each site correspond to the positions of the amino acid with KM114057 (the earliest reference strain of sub-genotype D3). Recombinant regions associated with the RF-Z and RF-AB sequences are highlighted by black boxes. The 6155–7297 nt region in the RF-AA sequence is highlighted by an orange box. The VP1 surface loops are shaded in grey.

To further investigate the amino acid mutation characteristics of the RF-Z, RF-AA, and RF-AB sequences, we compared the amino acid mutations of the eight CVA6 variants with KM114057 (the earliest reference strain of sub-genotype D3) (Fig. 5). Compared to KM114057, the RF-Z sequence exhibits amino acid mutations at 44 positions, the RF-AA sequence at 39 positions, and all RF-AB sequences share 41 common amino acid mutations. These mutations are distributed across the entire genome, suggesting that the RF-Z, RF-AA, and RF-AB variants evolved differently during transmission. The VP1 region plays a crucial role in immune evasion for enteroviruses. Six amino acid substitutions were identified in the RF-Z sequence (A5T, I20V, S27N, V30A, S137D, and A176S), with S137D located in the surface loop DE. The RF-AA sequence exhibited seven mutations (A5T, S13G, S27N, V30A, S137N, V242I, and T283A), including S137N in the DE loop and V242I in the HI loop. In the six RF-AB sequences, five variant residues were identified (A5T, S27N, V30A, D138N, and T283A), with the D138N mutation in CVA6/C-Tan/BJ/202316 mapping to the DE loop.

In the RF-Z and RF-AB recombinant variants, the number of amino acid mutations in the recombination regions showed a significant difference compared to the non-recombination regions (*P* < .001; Supplementary Table S8), suggesting that recombination in CVA6 introduces a higher frequency of amino acid changes. In the recombinant region of RF-Z, twenty-one amino acid mutations were identified, and RF-AB exhibited a common set of twenty-one amino acid mutations. Additionally, twelve amino acid mutations were identified in the 6155–7297 nt region of RF-AA.

### Global evolutionary dynamics of Coxsackievirus A6

**Figure 6:**
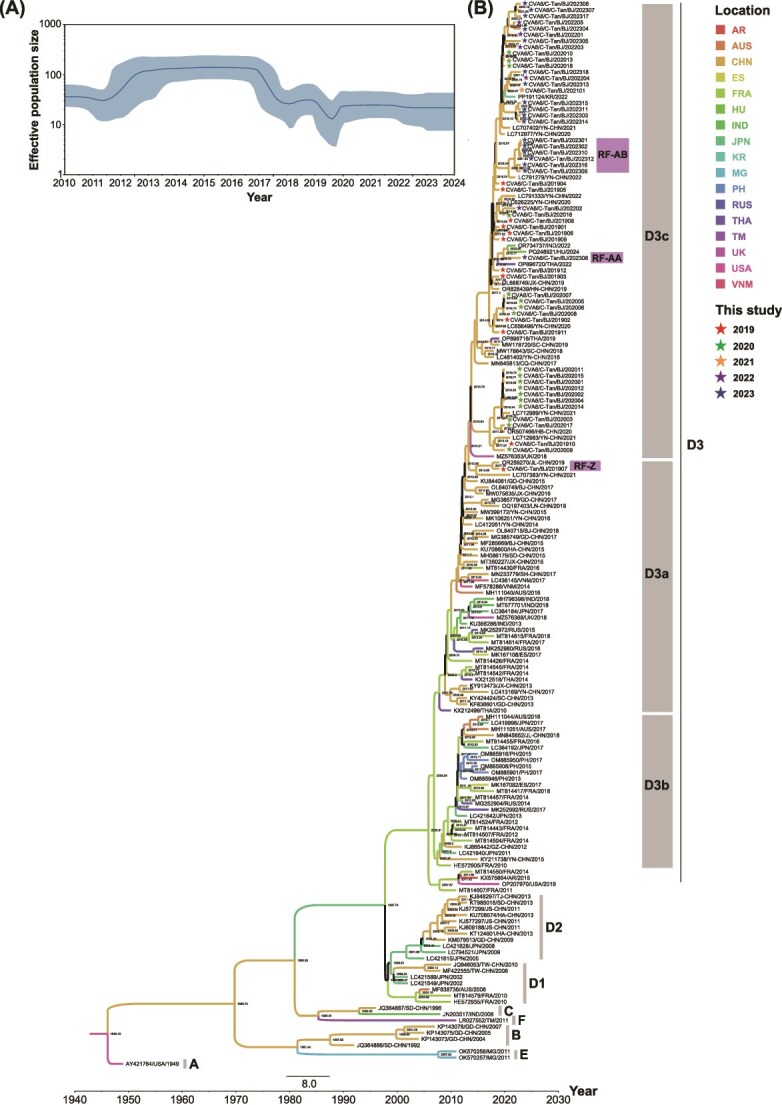
Global evolutionary dynamics of CVA6. (A) Bayesian skyline plot of the CVA6 VP1 region sequence, reflecting the relative genetic diversity of CVA6 from 2010 to 2024. The *X*-axis represents the time scale (in year), and the *Y*-axis shows the effective population size. The solid line is the estimated mean, and the blue shadow is the 95% highest posterior density interval. (B) The MCC phylogenetic tree generated using the MCMC method based on complete VP1 sequences of 172 CVA6. The colour of the branches represents the geographical location of sequences.

The evolutionary dynamics of CVA6 were reconstructed by analysing 172 complete VP1 sequences obtained from Sequence Library 1. The Bayesian skyline plot (Fig. 6A) shows that the effective population size of CVA6 increased rapidly from 2011 to 2013, reaching a relatively maximum size, which remained stable without significant fluctuations until 2017. However, a decline in effective population size was observed between 2017 and 2019, followed by a brief period of stability. Subsequently, the effective population size began to rise again after a short decline between 2019 and 2020, remaining stable with no significant fluctuations thereafter. This trend suggests that CVA6 has evolved at a relatively stable rate over the past 4 years.

The MCC tree was constructed using the MCMC method, which revealed that the average nucleotide substitution rate in the VP1 region of CVA6 was estimated to be 5.24 × 10^−3^ substitutions/site/year (95% highest probability density (HPD): 4.65 × 10^−3^ to 5.81 × 10^−3^). The most recent common ancestor (tMRCA) of CVA6 was estimated to have emerged in February 1946 (95% HPD: April 1939 to December 1948). For the D3c branch defined in this study, the tMRCA was estimated to have originated in August 2013 (95% HPD: May 2012 to September 2014). Furthermore, three RFs, namely RF-Z, RF-AA, and RF-AB, emerged in the D3a and D3c branches. A recombination event that led to the emergence of the RF-Z variant occurred between 2017 and 2019. The RF-AA variant, which emerged in 2023, likely resulted from recombination events that occurred between 2019 and 2023. Similarly, the RF-AB variants identified in 2023 underwent recombination between 2021 and 2023 (Fig. 6B).

## Discussion

Since the first reported outbreak of HFMD caused by CVA6 in Finland in 2008, CVA6-associated HFMD has rapidly spread across multiple countries and regions, leading to recurrent epidemics. Several provinces in China have also reported HFMD outbreaks attributed to CVA6 infections (Wang and Zhang 2023, Yang et al. 2023a, Chen et al. 2024). Infections with CVA6 often present with clinical features that distinguish them from those caused by other enteroviruses, such as CV-A16 and EV-A71, including desquamation, nail shedding, and extensive severe rashes (Deng et al. 2020, Justino et al. 2020, Zhao et al. 2020). Given the high incidence of CVA6-related diseases and their unique clinical manifestations, strengthening surveillance and implementing control measures for CVA6 infections are crucial. To this end, we performed whole-genome sequencing of CVA6 from patients in Beijing between 2019 and 2023 due to HFMD. We further analysed the phylogenetic characteristics, recombination diversity, and evolutionary dynamics of the CVA6 strains circulating in Beijing.

Song et al. previously classified the CVA6 D3 sub-genotype and further subdivided it into two branches, D3a and D3b, with an average genetic distance of 6% between them (Song et al. 2017). Recent CVA6 sequences have been classified into the sub-genotype D3, showing a noticeable increase in genetic divergence compared to earlier sequences. Furthermore, a gradual shift in the amino acid at position VP1-283 from Thr to Ala has been observed over recent years (Supplementary Fig. S3). To explore this further, 54 newly sequenced strains and 4947 high-quality sequences were incorporated to construct a ML tree (Supplementary Fig. S1). The analysis revealed that sequences with the VP1-283A mutation formed a distinct cluster, designated as the D3c branch, with 95.9% of the sequences exhibiting this mutation (Fig. 1C). The classification of the D3 sub-genotype followed the phylogenetic topology established by Song et al., incorporating the VP1 T283A mutation as an additional molecular marker and expanding the sample size to ensure methodological reproducibility. However, within the D3c branch, 33 sequences clustered together, exhibiting Thr at position VP1-283 instead of Ala. Phylogenetic analysis revealed that these sequences grouped with those carrying the VP1-283A variant, and the topology supported their classification within the D3c branch. The collection of these VP1-283T sequences between 2015 and 2018 suggests that strains with both VP1-283T and VP1-283A coexisted during the early formation of the D3c branch.

The global transmission patterns of the D3c branch reveal distinct epidemiological differences. The high prevalence of this branch in China (95.2%), the primary endemic region, is likely attributed to the large population, extensive domestic transportation network, and a well-established viral surveillance system. In contrast, the sporadic cases observed in Asian countries outside China, as well as in European and Oceanian regions, may be the result of viral introduction through international travel. Future research should incorporate multi-source data, including spatiotemporal dynamics of viral genomes, population migration patterns, and regional environmental factors, to provide a comprehensive analysis of the transmission characteristics of the D3c branch.

VP1-283 is located at the C-terminus of CVA6 VP1 protein. This site is involved in the formation of the conformational epitope of CVA6 (Liping et al. 2020), indicating that mutations at this position could influence the interaction of the virus with antibodies, potentially affecting the host’s antiviral response. Chen et al. identified six amino acid mutations in the CVA6 VP1 protein (V174I, T283A, A5T, V30A, S137N, and I242V) between 2010 and 2017, which may have contributed to large-scale outbreaks of CVA6 in Guangxi, China (Chen et al. 2019). The capsid proteins of enteroviruses typically contain key amino acid residues that play crucial roles in viral infection. For instance, an amino acid mutation at the VP2-142 site of CVA10 can inhibit viral binding to the KREMEN1 receptor, thereby enhancing the survival rate of neonatal mice (Li et al. 2023). Therefore, whether the CVA6 VP1-T283A amino acid mutation plays a role in viral infections remained to be determined and should be further validated through reverse genetics and site-directed mutagenesis.

Similar to the emergence of the SARS-CoV-2 variant carrying the D614G mutation in the spike protein, which became the most prevalent during the global pandemic (Korber et al. 2020), the CVA6 D3c branch, with the VP1-T283A mutation in 95.9% of sequences, has emerged as the globally dominant strain. Temporal dynamics analysis reveals a consistent increase in the proportion of the D3c branch. Therefore, several strategies for surveillance and prevention are proposed. Firstly, the CVA6 VP1-T283A mutation should be integrated as a key molecular marker in routine surveillance systems. Additionally, a standardized platform for neutralizing antibody assessments, utilizing reverse genetics technology, should be established to evaluate the immune evasion potential of variants. Finally, integrating viral genomic data, clinical characteristics, and epidemiological information provides scientific evidence to support the development of vaccines and antibody therapies.

During HFMD outbreaks, the co-circulation of multiple pathogens creates a favourable environment for recombination events among enteroviruses. Recombination is a common phenomenon that enables enteroviruses to acquire evolutionary diversity and new phenotypic traits (Andrés et al. 2019). Our study identified recombination events between CVA6 and other enterovirus A (EV-A) types, resulting in the emergence of new RFs, including RF-Z, RF-AA, and RF-AB, which reflect an increase in viral diversity. Notably, the RF-AA sequence formed an independent lineage. We were unable to find any sequences in the GenBank database that closely match the 6156–7321 nt region of the RF-AA sequence, so we conducted phylogenetic analysis on all sites and synonymous sites only within the 6155–7297 nt region and excluded the possibility of convergent evolution causing this phenomenon. This region, located within the 3D region and the 3′ UTR, both known as recombination hotspots in enterovirus, led us to hypothesize that the 6156–7321 nt region of the RF-AA sequence was likely acquired through recombination. Future studies should design specific primers targeting this region to further investigate its prevalence in different populations.

CVA6 exhibits a high frequency of genetic recombination, which can alter its virulence and facilitate the rapid evolution of other traits, posing a significant threat to public health (Wang and Wen 2024). A study conducted in Hong Kong identified recombinant CVA6 strains incorporating the 3D region from EV-A71, which were responsible for causing acute encephalitis in children (Lau et al. 2018). A systematic analysis of global CVA6 recombinants has revealed that different RFs may impact disease outcomes. Specifically, compared to RF-A, infections caused by RF-J are associated with more extensive rashes, while RF-L infections are more likely to lead to severe cases of HFMD (Song et al. 2020). We also identified recombination events between CVA6 and other EV-A species, resulting in the emergence of novel RFs, including RF-Z, RF-AA, and RF-AB. However, due to the lack of detailed clinical data, the pathogenic differences among these novel RFs remain unclear. Future studies should focus on building comprehensive clinical databases for recombinant strains, developing standardized animal models for comparative pathogenicity analysis, and employing multi-omics methods to explore the molecular relationships between recombination mechanisms and clinical manifestations.

The ORF region of the 54 CVA6 sequences in this study exhibited the highest nucleotide identity (96.05%–99.74%) with sequences obtained from various regions in China (including Beijing, Henan, Jilin, Yunnan, Chongqing, and Heilongjiang), as well as from neighbouring countries such as South Korea and Japan, all of which were classified as sub-genotype D3. This suggests that the CVA6 strains circulating in Beijing have not evolved independently but have co-evolved and co-circulated with isolates from other regions of China and neighbouring countries. This phenomenon is likely driven by population movement, which facilitates cross-border and cross-provincial transmission. Previous research has identified the BC, DE, EF, and HI surface loops within the VP1 capsid protein of CVA6 as putative neutralizing epitopes (Xu et al. 2017). In this study, we observed that the RF-Z and RF-AA sequences exhibited S137D and S137N mutations in the DE loop, respectively. The RF-AA sequence also carried a V242I mutation in the HI loop. Notably, the CVA6/C-Tan/BJ/202316 exhibited a D138N mutation in the DE loop, a site confirmed as a critical residue for the neutralizing epitope of the 1D5 monoclonal antibody (Xu et al. 2017). Furthermore, four amino acid mutations in the CVA6 VP1 protein (A5T, V30A, S137N, and T283A) were identified, which previous studies have suggested may be associated with the CVA6 epidemic in Guangxi Province (Chen et al. 2019). The analysis of amino acid mutations across the RF-Z and RF-AB sequences revealed a significant difference in the number of mutations between recombination and non-recombination regions. These findings suggest that recombination in CVA6 introduces more amino acid changes, which could be closely associated with the viral pathogenicity.

In summary, we established a comprehensive CVA6 VP1 sequence dataset consisting of 4736 global D3 sub-genotype sequences. Using this dataset, we defined a globally prevalent D3c evolutionary branch and identified its key amino acid mutation, T283A. Recombination analysis uncovered three novel RFs: RF-Z (1, 1.9%), RF-AA (1, 1.9%), and RF-AB (6, 11.1%). These findings provide important molecular epidemiological data on the evolutionary dynamics and genetic recombination of CVA6, enhancing our understanding of its genetic diversity and transmission patterns, and offering a solid foundation for future surveillance.

## Supplementary Material

Clean_Supplementary_Material_veaf036

## Data Availability

All sequences used in this study have been submitted to the National Genomics Data Center (NGDC) under accession numbers C_AA103342.1 to C_AA103395.1. All datasets and ML tree files are available at FigShare: https://doi.org/10.6084/m9.figshare.28836407.
