# Supplementary material for: Transition of D3c branch and novel recombination events contribute to the diversity of Coxsackievirus A6 in Beijing, China, from 2019 to 2023
Source: Virus Evol. 2025 May 11;11(1):veaf036. doi: 10.1093/ve/veaf036 (PMC12202042; doi:10.1093/ve/veaf036)
Supplement: Clean_Supplementary_Material_veaf036 [file clean_supplementary_material_veaf036.docx]

**Supplementary material**

**Detailed methods: Dataset construction**

A total of 4,972 CVA6 VP1 sequences were retrieved from GenBank (as of October 31, 2024). After excluding sequences with more than 5% ambiguous bases (denoted as "N"), incomplete or inaccurate sequences, 4,947 high-quality sequences were retained. To reduce sequence redundancy, clustering analysis was performed using a USEARCH tool with a 96% nucleotide identity threshold, resulting in the selection of 122 representative sequences. These 122 sequences were then combined with the 54 newly sequenced CVA6 VP1 sequences. TempEst (v1.5.3) was used to assess the correlation between nucleotide sequence divergence and sampling time. Four sequences with significant temporal bias were excluded, resulting in Sequence Library 1, which contains 172 VP1 sequences (**Table S2**).

To further analyze the molecular evolutionary characteristics of CVA6, datasets were constructed for both global and Beijing D3 sub-genotype sequences. The 54 new sequences were combined with the 4,947 sequences to construct a maximum likelihood (ML) tree (**Fig. S1**). Sequences from the D3a (2,742 sequences), D3b (473 sequences), and D3c (1,521 sequences) branches were selected to form Sequence Library 2 (**Table S3**). Additionally, 317 sequences from Beijing were selected from the 4,947 available global sequences. These were combined with the 54 new sequences and 43 reference sequences to construct another ML tree (**Fig. S2**). Sequences from the D3a (157 sequences), D3b (12 sequences), and D3c (192 sequences) branches were selected to form Sequence Library 3 (**Table S4**).

Furthermore, complete genome and CDS region sequences for CVA2 (106 sequences), CVA4 (183 sequences), CVA6 (1,055 sequences), and CVA8 (33 sequences) were retrieved from GenBank as of February 15, 2025, to identify parental sequences for the RF-AA analysis.

All datasets and ML tree files are available at FigShare: https://doi.org/10.6084/m9.figshare.28836407.

**
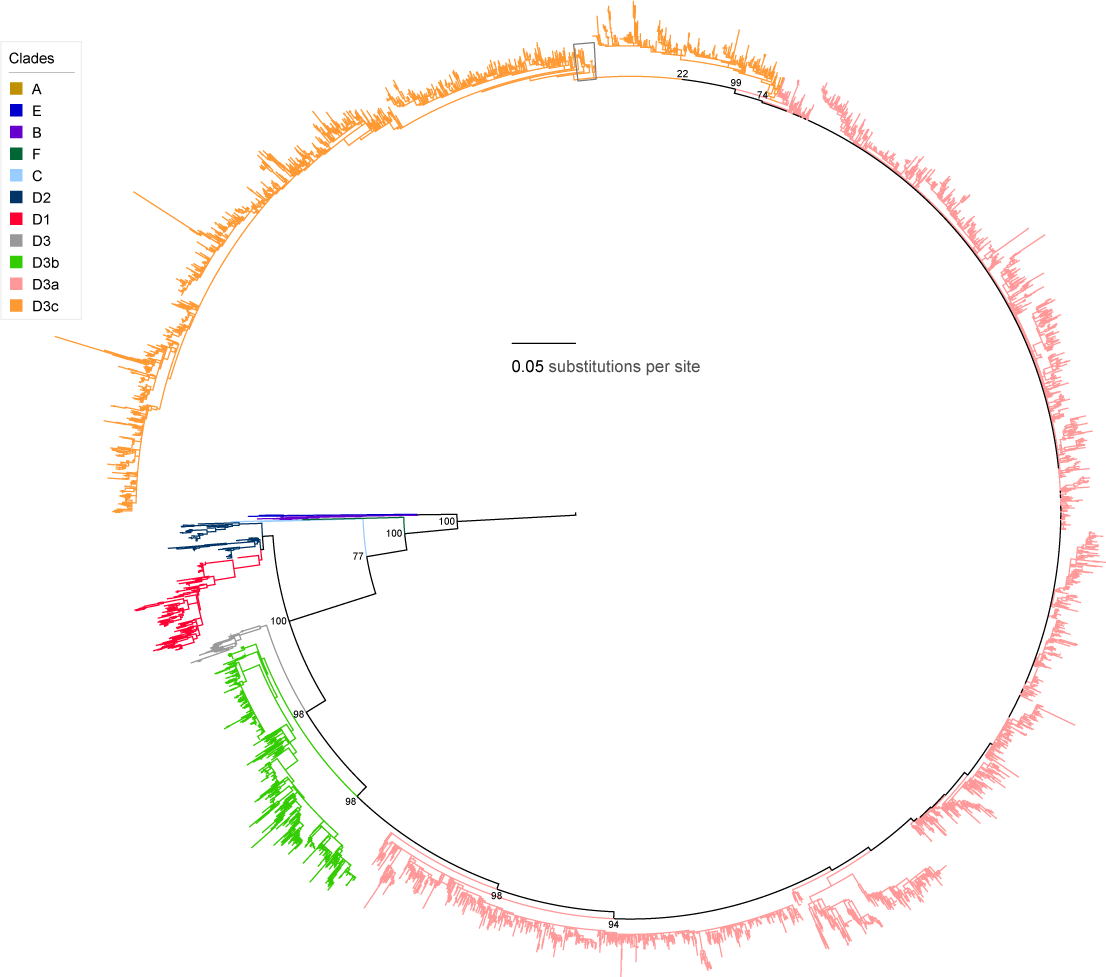
**

**Figure S1.** Phylogenetic analysis of VP1 sequences (nucleotide positions 2,441–3,355, 915 nt, relative to AY421764) using 5,001 CVA6 VP1 sequences. Maximum likelihood (ML) tree was constructed with 1,000 bootstrap replicates to assess the robustness of the groupings. The 33 sequences highlighted in gray boxes exhibit threonine (Thr) at amino acid position 283, instead of alanine (Ala).


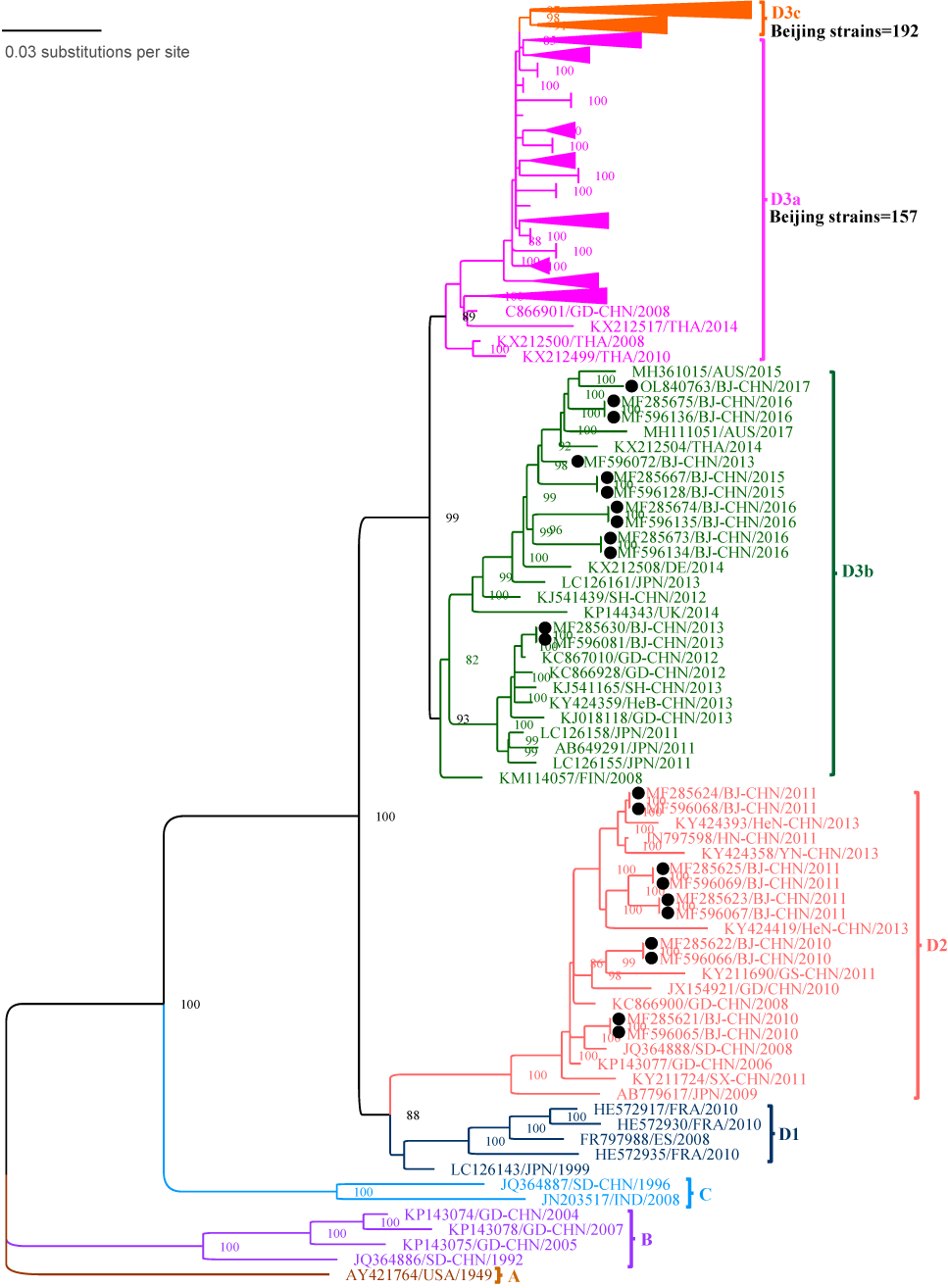


**Figure S2.** Phylogenetic analysis of VP1 sequences (nucleotide positions 2,441–3,355, 915 nt, relative to AY421764) using 414 CVA6 VP1 sequences, including 371 sequences from Beijing and 43 reference sequences. Maximum likelihood (ML) tree was constructed with 1,000 bootstrap replicates to assess the robustness of the groupings. The 22 Beijing sequences are marked with black circles.

**
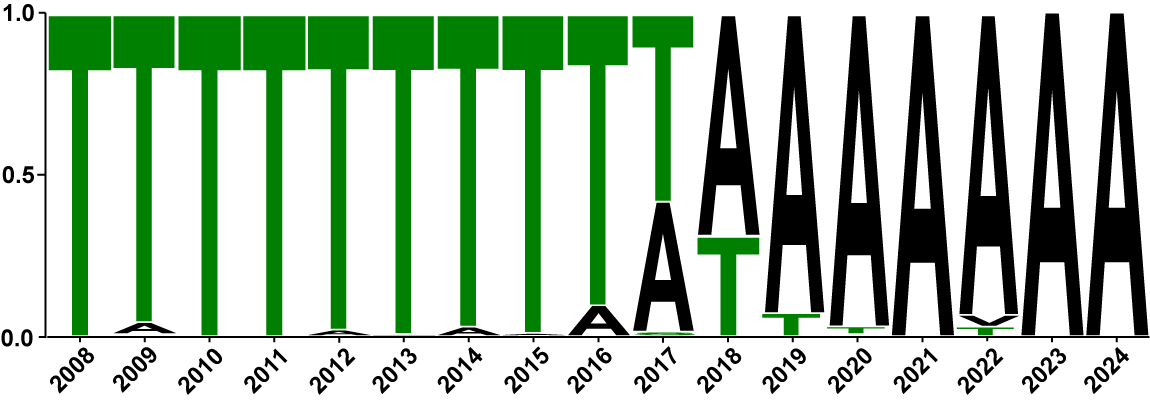
**

**Figure S3.** Amino acid composition at position VP1-283 from 2008 to 2024, based on a total of 4,736 CVA6 sequences from Sequence Library 2.

**
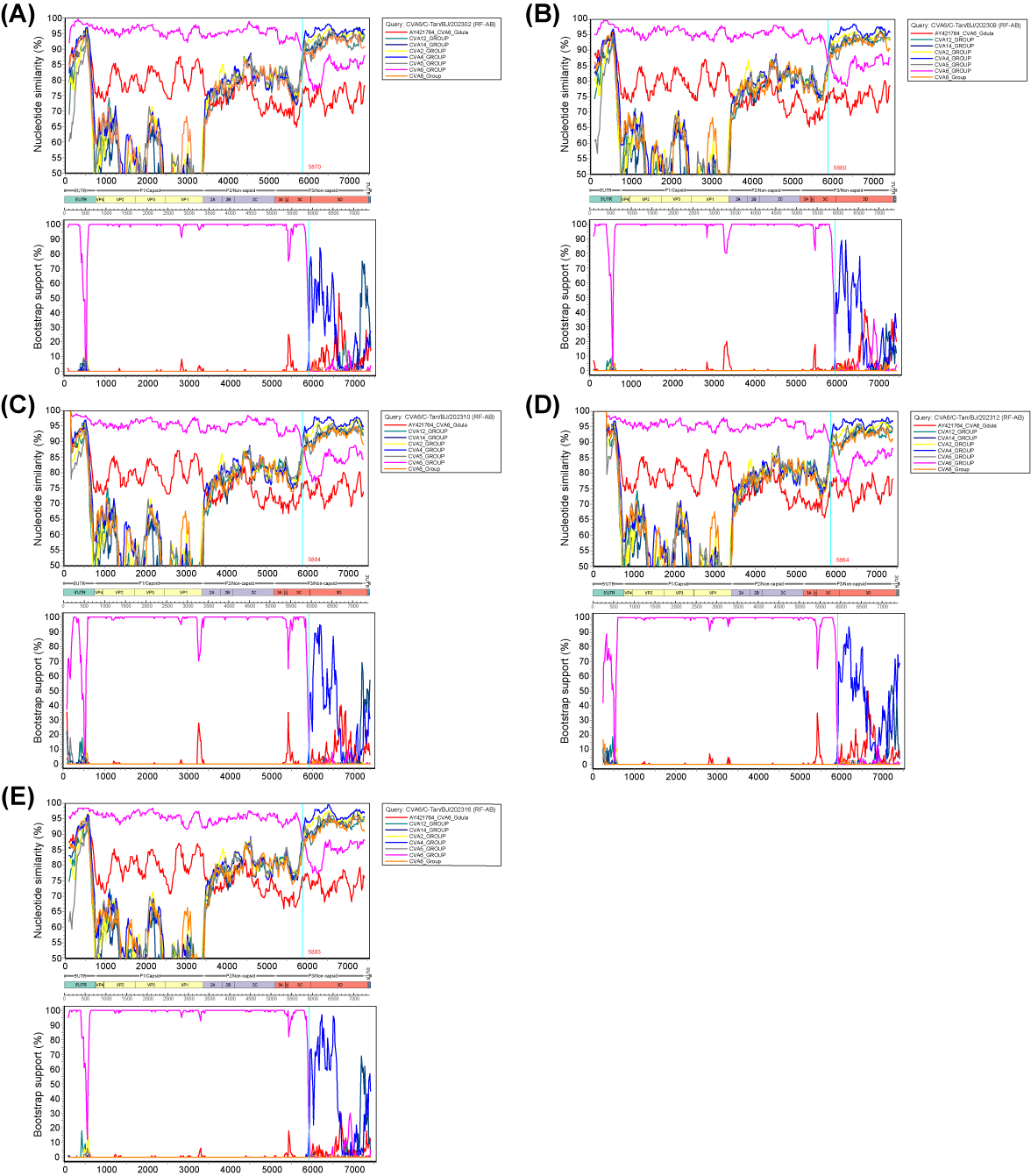
**

**Figure S4.** Recombination events in the five sequences. (A) CVA6/C-Tan/BJ/202302, (B) CVA6/C-Tan/BJ/202309, (C) CVA6/C-Tan/BJ/202310, (D) CVA6/C-Tan/BJ/202312, and (E) CVA6/C-Tan/BJ/202316 are shown using similarity plots and bootscanning analyses with a sliding window of 200 nt and a step size of 20 nt.

**Table S1.** Information on the primers for amplifying regions of CVA6.

| **Primer** | **Position (nt)** | **Primer sequence (5'–3')** | **Orientation** |
| --- | --- | --- | --- |
| CVA6-509F | 489-509 | AGCACATGCYCTCAATCCAG | Forward |
| CVA6-964R | 964-985 | ACTATAACCGCACGCCTCAAC | Reverse |
| CVA6-891F | 868-891 | GCTAGTAGACAGGACTTTGCACA | Forward |
| CVA6-1336R | 1336-1356 | GCAGCAACCACAAATTCGGG | Reverse |
| CVA6-1265F | 1242-1265 | CCAATTCCACTACTTGTACCGCT | Forward |
| CVA6-1702R | 1702-1726 | CCCTTGTTTTATTGCTTGTCGGAG | Reverse |
| CVA6-1623F | 1601-1623 | TGGTYGTGATCCCTATTAGCCC | Forward |
| CVA6-2080R | 2080-2104 | CCCTGTAGCCATAAAAGAACCTGT | Reverse |
| CVA6-1996F | 1976-1996 | AAGTAGACCCTGGGCGCAAT | Forward |
| CVA6-2459R | 2459-2481 | CTCACAGCGCTTTCTACTGCAT | Reverse |
| CVA6-2385F | 2363-2385 | CTCTTGGAGCAGCACAGAGAAA | Forward |
| CVA6-2825R | 2825-2848 | GTTGGACACAAAAGTGAACTCGG | Reverse |
| CVA6-2741F | 2719-2741 | GACTCGGGCACTARCTTGGATG | Forward |
| CVA6-3212R | 3212-3234 | TGGGATCGAAGGGGTCTAGGTA | Reverse |
| CVA6-3136F | 3114-3136 | GATGGGCCATTTTGCYATCCGA | Forward |
| CVA6-3585R | 3585-3606 | AAGACGAGGCTGGGYTTAGAA | Reverse |
| CVA6-3512F | 3490-3512 | ACCACTGCTCAGGGATGTGATA | Forward |
| CVA6-3953R | 3953-3981 | CTGATCACTATGACTAATGCTGAGATCA | Reverse |
| CVA6-3882F | 3860-3882 | CTGATGCAGTRGCTAGGGAGGT | Forward |
| CVA6-4321R | 4321-4344 | ACGTTCCCAAACATTGCYTCAAG | Reverse |
| CVA6-4224F | 4197-4224 | GCTTAAGGAGAAAATTATACCAGCAGC | Forward |
| CVA6-4666R | 4666-4692 | GTGGATACCATCTGRCAAAAGAGTGA | Reverse |
| CVA6-4578F | 4554-4578 | CCACTCTAGTGTGTACTCACTTCC | Forward |
| CVA6-5053R | 5053-5075 | TTGTGTTTCCAATGGCAGACCT | Reverse |
| CVA6-4974F | 4953-4974 | ACTAGTGTGTGGGAAGGCCAT | Forward |
| CVA6-5423R | 5423-5445 | AGGGATAGGGCAAAATCAAGGC | Reverse |
| CVA6-5334F | 5308-5334 | TCACTGGTGTATGTTATCTACAAGCT | Forward |
| CVA6-5791R | 5791-5813 | GGGTBGGCTTTCCACTGAGATT | Reverse |
| CVA6-5696F | 5671-5696 | ATCACCAAGTTCATTCCAGAAAACA | Forward |
| CVA6-6133R | 6133-6162 | GTGTTTCCTACATACTTRGAGAACAATGC | Reverse |
| CVA6-6033F | 6013-6033 | ATCAAYGGGCCAACTCRCAC | Forward |
| CVA6-6512R | 6512-6535 | GGCCATTCTGAGGTARACTGAGT | Reverse |
| CVA6-6402F | 6373-6402 | GATGTGAGTAAGATGAAGTTYTACATGGA | Forward |
| CVA6-6844R | 6844-6873 | TTRTTAATCATTGAGTTGAARATGGATGT | Reverse |
| CVA6-6709F | 6688-6709 | AGYCCAGTTTGGTTYAGAGCA | Forward |
| CVA6-7190R | 7190-7212 | CGCACGTGATCTTGAGTRTTGC | Reverse |

**Table S2.** Information on 172 CVA6 for phylogenetic analysis.

| **Strain name/GenBank No.** | **Country** | **Isolated year** | **Genotype/Sub-genotype** | **Source** |
| --- | --- | --- | --- | --- |
| AY421764 | America | 1949 | A | GenBank |
| JQ364886 | China | 1992 | B | GenBank |
| JQ364887 | China | 1996 | C | GenBank |
| LC421549 | Japan | 2002 | D1 | GenBank |
| LC421589 | Japan | 2002 | D1 | GenBank |
| KP143073 | China | 2004 | B | GenBank |
| KP143075 | China | 2005 | B | GenBank |
| LC421615 | Japan | 2005 | D2 | GenBank |
| MF838736 | Australia | 2006 | D1 | GenBank |
| KP143078 | China | 2007 | B | GenBank |
| JN203517 | India | 2008 | C | GenBank |
| MF422555 | China | 2008 | D1 | GenBank |
| LC421628 | Japan | 2008 | D2 | GenBank |
| KM079513 | China | 2009 | D2 | GenBank |
| LC794521 | Japan | 2009 | D2 | GenBank |
| HE572935 | France | 2010 | D1 | GenBank |
| JQ946053 | China | 2010 | D1 | GenBank |
| MT814579 | France | 2010 | D1 | GenBank |
| KX212499 | Thailand | 2010 | D3a | GenBank |
| HE572905 | France | 2010 | D3b | GenBank |
| KJ577297 | China | 2011 | D2 | GenBank |
| KJ577299 | China | 2011 | D2 | GenBank |
| KJ609188 | China | 2011 | D2 | GenBank |
| MT814607 | France | 2011 | D3 | GenBank |
| LC421640 | Japan | 2011 | D3b | GenBank |
| OK570257 | Madagascar | 2011 | E | GenBank |
| OK570258 | Madagascar | 2011 | E | GenBank |
| LR027552 | Turkmenistan | 2011 | F | GenBank |
| KJ865442 | China | 2012 | D3b | GenBank |
| MT814507 | France | 2012 | D3b | GenBank |
| MT814524 | France | 2012 | D3b | GenBank |
| KJ848297 | China | 2013 | D2 | GenBank |
| KT124601 | China | 2013 | D2 | GenBank |
| KT985016 | China | 2013 | D2 | GenBank |
| KU708574 | China | 2013 | D2 | GenBank |
| KF836601 | China | 2013 | D3a | GenBank |
| KU366286 | India | 2013 | D3a | GenBank |
| KY424424 | China | 2013 | D3a | GenBank |
| KY913473 | China | 2013 | D3a | GenBank |
| LC421642 | Japan | 2013 | D3b | GenBank |
| OM885946 | Philippines | 2013 | D3b | GenBank |
| MT814550 | France | 2014 | D3 | GenBank |
| KX212518 | Thailand | 2014 | D3a | GenBank |
| LC412051 | China | 2014 | D3a | GenBank |
| MF578288 | Vietnam | 2014 | D3a | GenBank |
| MT814428 | France | 2014 | D3a | GenBank |
| MT814542 | France | 2014 | D3a | GenBank |
| MT814545 | France | 2014 | D3a | GenBank |
| MG252904 | Russia | 2014 | D3b | GenBank |
| MT814443 | France | 2014 | D3b | GenBank |
| MT814457 | France | 2014 | D3b | GenBank |
| MT814504 | France | 2014 | D3b | GenBank |
| KX575864 | Argentina | 2015 | D3 | GenBank |
| KU708600 | China | 2015 | D3a | GenBank |
| KU844081 | China | 2015 | D3a | GenBank |
| MF285669 | China | 2015 | D3a | GenBank |
| MH086179 | China | 2015 | D3a | GenBank |
| MK252972 | Russia | 2015 | D3a | GenBank |
| MT350227 | China | 2015 | D3a | GenBank |
| MW399172 | China | 2015 | D3a | GenBank |
| KY211738 | China | 2015 | D3b | GenBank |
| OM885908 | Philippines | 2015 | D3b | GenBank |
| OM885916 | Philippines | 2015 | D3b | GenBank |
| MH111040 | Australia | 2016 | D3a | GenBank |
| MK106251 | China | 2016 | D3a | GenBank |
| MK252980 | Russia | 2016 | D3a | GenBank |
| MT814430 | France | 2016 | D3a | GenBank |
| MW075635 | China | 2016 | D3a | GenBank |
| MH111044 | Australia | 2016 | D3b | GenBank |
| MT814455 | France | 2016 | D3b | GenBank |
| LC364184 | Japan | 2017 | D3a | GenBank |
| LC413169 | China | 2017 | D3a | GenBank |
| LC438145 | Vietnam | 2017 | D3a | GenBank |
| MG385749 | China | 2017 | D3a | GenBank |
| MG385779 | China | 2017 | D3a | GenBank |
| MK167108 | Spain | 2017 | D3a | GenBank |
| MN233779 | China | 2017 | D3a | GenBank |
| MT814614 | France | 2017 | D3a | GenBank |
| OL840749 | China | 2017 | D3a | GenBank |
| LC364192 | Japan | 2017 | D3b | GenBank |
| LC419996 | Japan | 2017 | D3b | GenBank |
| MH111051 | Australia | 2017 | D3b | GenBank |
| MK167082 | Spain | 2017 | D3b | GenBank |
| MK252992 | Russia | 2017 | D3b | GenBank |
| OM885901 | Philippines | 2017 | D3b | GenBank |
| OM885950 | Philippines | 2017 | D3b | GenBank |
| MN845813 | China | 2017 | D3c | GenBank |
| MH796398 | India | 2018 | D3a | GenBank |
| MT577701 | India | 2018 | D3a | GenBank |
| MT814615 | France | 2018 | D3a | GenBank |
| MZ576368 | United Kingdom | 2018 | D3a | GenBank |
| OL840715 | China | 2018 | D3a | GenBank |
| MN845852 | China | 2018 | D3b | GenBank |
| MT814417 | France | 2018 | D3b | GenBank |
| LC481402 | China | 2018 | D3c | GenBank |
| MW178643 | China | 2018 | D3c | GenBank |
| MZ576353 | United Kingdom | 2018 | D3c | GenBank |
| OP207970 | America | 2019 | D3 | GenBank |
| OQ197403 | China | 2019 | D3a | GenBank |
| CVA6/C-Tan/BJ/201901 | China | 2019 | D3c | This study |
| CVA6/C-Tan/BJ/201902 | China | 2019 | D3c | This study |
| CVA6/C-Tan/BJ/201903 | China | 2019 | D3c | This study |
| CVA6/C-Tan/BJ/201904 | China | 2019 | D3c | This study |
| CVA6/C-Tan/BJ/201905 | China | 2019 | D3c | This study |
| CVA6/C-Tan/BJ/201906 | China | 2019 | D3c | This study |
| CVA6/C-Tan/BJ/201907 | China | 2019 | D3c | This study |
| CVA6/C-Tan/BJ/201908 | China | 2019 | D3c | This study |
| CVA6/C-Tan/BJ/201909 | China | 2019 | D3c | This study |
| CVA6/C-Tan/BJ/201910 | China | 2019 | D3c | This study |
| CVA6/C-Tan/BJ/201911 | China | 2019 | D3c | This study |
| CVA6/C-Tan/BJ/201912 | China | 2019 | D3c | This study |
| MW178720 | China | 2019 | D3c | GenBank |
| OL688749 | China | 2019 | D3c | GenBank |
| OP896716 | Thailand | 2019 | D3c | GenBank |
| OR259270 | China | 2019 | D3c | GenBank |
| OR828439 | China | 2019 | D3c | GenBank |
| CVA6/C-Tan/BJ/202001 | China | 2020 | D3c | This study |
| CVA6/C-Tan/BJ/202002 | China | 2020 | D3c | This study |
| CVA6/C-Tan/BJ/202003 | China | 2020 | D3c | This study |
| CVA6/C-Tan/BJ/202004 | China | 2020 | D3c | This study |
| CVA6/C-Tan/BJ/202005 | China | 2020 | D3c | This study |
| CVA6/C-Tan/BJ/202006 | China | 2020 | D3c | This study |
| CVA6/C-Tan/BJ/202007 | China | 2020 | D3c | This study |
| CVA6/C-Tan/BJ/202008 | China | 2020 | D3c | This study |
| CVA6/C-Tan/BJ/202009 | China | 2020 | D3c | This study |
| CVA6/C-Tan/BJ/202010 | China | 2020 | D3c | This study |
| CVA6/C-Tan/BJ/202011 | China | 2020 | D3c | This study |
| CVA6/C-Tan/BJ/202012 | China | 2020 | D3c | This study |
| CVA6/C-Tan/BJ/202013 | China | 2020 | D3c | This study |
| CVA6/C-Tan/BJ/202014 | China | 2020 | D3c | This study |
| CVA6/C-Tan/BJ/202015 | China | 2020 | D3c | This study |
| CVA6/C-Tan/BJ/202016 | China | 2020 | D3c | This study |
| CVA6/C-Tan/BJ/202017 | China | 2020 | D3c | This study |
| CVA6/C-Tan/BJ/202018 | China | 2020 | D3c | This study |
| LC626225 | China | 2020 | D3c | GenBank |
| LC656496 | China | 2020 | D3c | GenBank |
| LC712977 | China | 2020 | D3c | GenBank |
| OR507466 | China | 2020 | D3c | GenBank |
| CVA6/C-Tan/BJ/202101 | China | 2021 | D3c | This study |
| LC707383 | China | 2021 | D3c | GenBank |
| LC707402 | China | 2021 | D3c | GenBank |
| LC712983 | China | 2021 | D3c | GenBank |
| LC712989 | China | 2021 | D3c | GenBank |
| CVA6/C-Tan/BJ/202201 | China | 2022 | D3c | This study |
| CVA6/C-Tan/BJ/202202 | China | 2022 | D3c | This study |
| CVA6/C-Tan/BJ/202203 | China | 2022 | D3c | This study |
| CVA6/C-Tan/BJ/202204 | China | 2022 | D3c | This study |
| CVA6/C-Tan/BJ/202205 | China | 2022 | D3c | This study |
| LC791279 | China | 2022 | D3c | GenBank |
| LC791333 | China | 2022 | D3c | GenBank |
| OP896720 | Thailand | 2022 | D3c | GenBank |
| OR734737 | India | 2022 | D3c | GenBank |
| PP191124 | South Korea | 2022 | D3c | GenBank |
| CVA6/C-Tan/BJ/202301 | China | 2023 | D3c | This study |
| CVA6/C-Tan/BJ/202302 | China | 2023 | D3c | This study |
| CVA6/C-Tan/BJ/202303 | China | 2023 | D3c | This study |
| CVA6/C-Tan/BJ/202304 | China | 2023 | D3c | This study |
| CVA6/C-Tan/BJ/202305 | China | 2023 | D3c | This study |
| CVA6/C-Tan/BJ/202306 | China | 2023 | D3c | This study |
| CVA6/C-Tan/BJ/202307 | China | 2023 | D3c | This study |
| CVA6/C-Tan/BJ/202308 | China | 2023 | D3c | This study |
| CVA6/C-Tan/BJ/202309 | China | 2023 | D3c | This study |
| CVA6/C-Tan/BJ/202310 | China | 2023 | D3c | This study |
| CVA6/C-Tan/BJ/202311 | China | 2023 | D3c | This study |
| CVA6/C-Tan/BJ/202312 | China | 2023 | D3c | This study |
| CVA6/C-Tan/BJ/202313 | China | 2023 | D3c | This study |
| CVA6/C-Tan/BJ/202314 | China | 2023 | D3c | This study |
| CVA6/C-Tan/BJ/202315 | China | 2023 | D3c | This study |
| CVA6/C-Tan/BJ/202316 | China | 2023 | D3c | This study |
| CVA6/C-Tan/BJ/202317 | China | 2023 | D3c | This study |
| CVA6/C-Tan/BJ/202318 | China | 2023 | D3c | This study |
| PQ248921 | Hungary | 2024 | D3c | GenBank |

**Table S3.** The 4,736 global CVA6 D3 sub-genotype sequences for phylogenetic analysis.

| **Year** | **Number of D3b (%)** | **Number of D3a (%)** | **Number of D3c (%)** | **Total** |
| --- | --- | --- | --- | --- |
| 2008 | 4 (66.7) | 2 (33.3) | 0 (0) | 6 |
| 2009 | 0 (0) | 27 (100) | 0 (0) | 27 |
| 2010 | 15 (13.8) | 94 (86.2) | 0 (0) | 109 |
| 2011 | 39 (21.0) | 147 (79.0) | 0 (0) | 186 |
| 2012 | 94 (26.6) | 259 (73.4) | 0 (0) | 353 |
| 2013 | 103 (13.1) | 685 (86.9) | 0 (0) | 788 |
| 2014 | 47 (11.4) | 364 (88.6) | 0 (0) | 411 |
| 2015 | 63 (12.0) | 444 (84.9) | 16 (3.1) | 523 |
| 2016 | 28 (10.2) | 222 (81.0) | 24 (8.8) | 274 |
| 2017 | 76 (11.5) | 315 (47.7) | 269 (40.8) | 660 |
| 2018 | 3 (0.6) | 157 (28.8) | 385 (70.6) | 545 |
| 2019 | 1 (0.4) | 18 (6.8) | 246 (92.8) | 265 |
| 2020 | 0 (0) | 4 (2.4) | 160 (97.6) | 164 |
| 2021 | 0 (0) | 3 (1.3) | 234 (98.7) | 237 |
| 2022 | 0 (0) | 1 (0.7) | 135 (99.3) | 136 |
| 2023 | 0 (0) | 0 (0) | 50 (100) | 50 |
| 2024 | 0 (0) | 0 (0) | 2 (100) | 2 |

**Table S4.** The 361 CVA6 D3 sub-genotype sequences from Beijing for phylogenetic analysis.

| **Year** | **Number of D3b (%)** | **Number of D3a (%)** | **Number of D3c (%)** | **Total** |
| --- | --- | --- | --- | --- |
| 2013 | 3 (11.1) | 24 (88.9) | 0 (0) | 27 |
| 2014 | 0 (0) | 37 (100) | 0 (0) | 37 |
| 2015 | 2 (4.2) | 40 (83.3) | 6 (12.5) | 48 |
| 2016 | 6 (16.7) | 26 (72.2) | 4 (11.1) | 36 |
| 2017 | 1 (2.8) | 18 (48.6) | 18 (48.6) | 37 |
| 2018 | 0 (0) | 8 (9.0) | 81 (91.0) | 89 |
| 2019 | 0 (0) | 4 (8.9) | 41 (91.1) | 45 |
| 2020 | 0 (0) | 0 (0) | 18 (100) | 18 |
| 2021 | 0 (0) | 0 (0) | 1 (100) | 1 |
| 2022 | 0 (0) | 0 (0) | 5 (100) | 5 |
| 2023 | 0 (0) | 0 (0) | 18 (100) | 18 |

**Table S5.** Epidemiological characteristics of 54 CVA6 sequences based on the ORF region.

| **Strain name** | **GenBase accession number** | **Specimen type** | **Nearest CVA6 strain in**  **ORF region (Accession No.)** | **Similarity with the**  **nearest CVA6**  **strain (%)** |
| --- | --- | --- | --- | --- |
| CVA6/C-Tan/BJ/201901 | C_AA103342.1 | Throat swab | YN-CHN/2018 (OL839942) | 98.79 |
| CVA6/C-Tan/BJ/201902 | C_AA103343.1 | Throat swab | CQ-CHN/2018 (MN845816) | 99.03 |
| CVA6/C-Tan/BJ/201903 | C_AA103344.1 | Throat swab | CQ-CHN/2018 (MN845816) | 98.82 |
| CVA6/C-Tan/BJ/201904 | C_AA103345.1 | Throat swab | YN-CHN/2018 (OL839938) | 98.86 |
| CVA6/C-Tan/BJ/201905 | C_AA103346.1 | Throat swab | JPN/2022 (LC789938) | 99.26 |
| CVA6/C-Tan/BJ/201906 | C_AA103347.1 | Throat swab | BJ-CHN/2019 (OL830027) | 99.62 |
| CVA6/C-Tan/BJ/201907 | C_AA103348.1 | Throat swab | JL-CHN/2019 (OR394968) | 98.46 |
| CVA6/C-Tan/BJ/201908 | C_AA103349.1 | Throat swab | YN-CHN/2018 (OL839938) | 98.62 |
| CVA6/C-Tan/BJ/201909 | C_AA103350.1 | Throat swab | BJ-CHN/2019 (OL830029) | 99.74 |
| CVA6/C-Tan/BJ/201910 | C_AA103351.1 | Throat swab | YN-CHN/2018 (OL839935) | 98.88 |
| CVA6/C-Tan/BJ/201911 | C_AA103352.1 | Throat swab | CQ-CHN/2018 (MN845816) | 98.62 |
| CVA6/C-Tan/BJ/201912 | C_AA103353.1 | Throat swab | YN-CHN/2018 (OL839942) | 98.80 |
| CVA6/C-Tan/BJ/202001 | C_AA103354.1 | Throat swab | HA-CHN/2023 (OR500231) | 99.30 |
| CVA6/C-Tan/BJ/202002 | C_AA103355.1 | Throat swab | HA-CHN/2023 (OR500231) | 99.36 |
| CVA6/C-Tan/BJ/202003 | C_AA103356.1 | Throat swab | HA-CHN/2023 (OR500231) | 99.33 |
| CVA6/C-Tan/BJ/202004 | C_AA103357.1 | Throat swab | HA-CHN/2023 (OR500231) | 99.29 |
| CVA6/C-Tan/BJ/202005 | C_AA103358.1 | Throat swab | CQ-CHN/2018 (MN845816) | 98.41 |
| CVA6/C-Tan/BJ/202006 | C_AA103359.1 | Throat swab | CQ-CHN/2018 (MN845816) | 98.44 |
| CVA6/C-Tan/BJ/202007 | C_AA103360.1 | Throat swab | CQ-CHN/2018 (MN845816) | 98.41 |
| CVA6/C-Tan/BJ/202008 | C_AA103361.1 | Throat swab | CQ-CHN/2018 (MN845816) | 98.40 |
| CVA6/C-Tan/BJ/202009 | C_AA103362.1 | Throat swab | YN-CHN/2018 (OL839935) | 98.47 |
| CVA6/C-Tan/BJ/202010 | C_AA103363.1 | Throat swab | JL-CHN/2022 (OR394975) | 99.05 |
| CVA6/C-Tan/BJ/202011 | C_AA103364.1 | Throat swab | HA-CHN/2023 (OR500231) | 99.33 |
| CVA6/C-Tan/BJ/202012 | C_AA103365.1 | Throat swab | HA-CHN/2023 (OR500231) | 99.46 |
| CVA6/C-Tan/BJ/202013 | C_AA103366.1 | Throat swab | JL-CHN/2022 (OR394975) | 99.03 |
| CVA6/C-Tan/BJ/202014 | C_AA103367.1 | Throat swab | HA-CHN/2023 (OR500231) | 99.30 |
| CVA6/C-Tan/BJ/202015 | C_AA103368.1 | Throat swab | HA-CHN/2023 (OR500231) | 99.30 |
| CVA6/C-Tan/BJ/202016 | C_AA103369.1 | Throat swab | YN-CHN/2018 (OL839938) | 98.59 |
| CVA6/C-Tan/BJ/202017 | C_AA103370.1 | Throat swab | BJ-CHN/2017 (OL830036) | 98.35 |
| CVA6/C-Tan/BJ/202018 | C_AA103371.1 | Throat swab | JL-CHN/2022 (OR394975) | 99.09 |
| CVA6/C-Tan/BJ/202101 | C_AA103372.1 | Throat swab | KR/2022 (PP191112) | 98.77 |
| CVA6/C-Tan/BJ/202201 | C_AA103373.1 | Throat swab | JL-CHN/2022 (OR394975) | 98.65 |
| CVA6/C-Tan/BJ/202202 | C_AA103374.1 | Throat swab | YN-CHN/2018 (OL839938) | 98.40 |
| CVA6/C-Tan/BJ/202203 | C_AA103375.1 | Throat swab | JL-CHN/2022 (OR394975) | 98.15 |
| CVA6/C-Tan/BJ/202204 | C_AA103376.1 | Throat swab | KR/2022 (PP191112) | 98.32 |
| CVA6/C-Tan/BJ/202205 | C_AA103377.1 | Throat swab | JL-CHN/2022 (OR394975) | 98.49 |
| CVA6/C-Tan/BJ/202301 | C_AA103378.1 | Throat swab | HA-CHN/2023 (OR500230) | 99.12 |
| CVA6/C-Tan/BJ/202302 | C_AA103379.1 | Vesicular fluid | HA-CHN/2023 (OR500230) | 99.14 |
| CVA6/C-Tan/BJ/202303 | C_AA103380.1 | Vesicular fluid | JL-CHN/2022 (OR394975) | 98.09 |
| CVA6/C-Tan/BJ/202304 | C_AA103381.1 | Vesicular fluid | JL-CHN/2022 (OR394975) | 98.32 |
| CVA6/C-Tan/BJ/202305 | C_AA103382.1 | Vesicular fluid | JL-CHN/2022 (OR394975) | 97.94 |
| CVA6/C-Tan/BJ/202306 | C_AA103383.1 | Vesicular fluid | JL-CHN/2022 (OR394975) | 98.27 |
| CVA6/C-Tan/BJ/202307 | C_AA103384.1 | Throat swab | JL-CHN/2022 (OR394975) | 98.24 |
| CVA6/C-Tan/BJ/202308 | C_AA103385.1 | Throat swab | HLJ-CHN/2018 (MN845848) | 96.05 |
| CVA6/C-Tan/BJ/202309 | C_AA103386.1 | Throat swab | HA-CHN/2023 (OR500230) | 98.44 |
| CVA6/C-Tan/BJ/202310 | C_AA103387.1 | Throat swab | HA-CHN/2023 (OR500230) | 99.09 |
| CVA6/C-Tan/BJ/202311 | C_AA103388.1 | Throat swab | KR/2022 (PP191112) | 98.06 |
| CVA6/C-Tan/BJ/202312 | C_AA103389.1 | Throat swab | HA-CHN/2023 (OR500230) | 98.99 |
| CVA6/C-Tan/BJ/202313 | C_AA103390.1 | Throat swab | KR/2022 (PP191112) | 98.09 |
| CVA6/C-Tan/BJ/202314 | C_AA103391.1 | Throat swab | KR/2022 (PP191112) | 98.00 |
| CVA6/C-Tan/BJ/202315 | C_AA103392.1 | Throat swab | KR/2022 (PP191112) | 97.91 |
| CVA6/C-Tan/BJ/202316 | C_AA103393.1 | Throat swab | HA-CHN/2023 (OR500230) | 98.62 |
| CVA6/C-Tan/BJ/202317 | C_AA103394.1 | Throat swab | JL-CHN/2022 (OR394975) | 98.38 |
| CVA6/C-Tan/BJ/202318 | C_AA103395.1 | Throat swab | KR/2022 (PP191113) | 98.21 |

**Table S6.** Geographic and temporal distribution of VP1 sequences in the CVA6 D3c branch.

| Region | Country | Numbers of sequences | | | | | | | | | | |
| --- | --- | --- | --- | --- | --- | --- | --- | --- | --- | --- | --- | --- |
|  |  | 2015 | 2016 | 2017 | 2018 | 2019 | 2020 | 2021 | 2022 | 2023 | 2024 | Total |
| Asia | China | 16 | 24 | 258 | 377 | 238 | 159 | 233 | 93 | 50 |  | 1448 |
|  | Japan |  |  |  |  | 2 |  | 1 | 4 |  |  | 7 |
|  | South Korea |  |  |  |  |  |  |  | 16 |  |  | 16 |
|  | India |  |  |  |  |  |  |  | 8 |  |  | 8 |
|  | Thailand |  |  |  |  | 6 | 1 |  | 13 |  |  | 20 |
|  | Vietnam |  |  | 6 |  |  |  |  |  |  |  | 6 |
| Europe | France |  |  | 2 | 2 |  |  |  |  |  |  | 4 |
|  | Hungary |  |  |  |  |  |  |  | 1 |  | 2 | 3 |
|  | Russia |  |  | 2 |  |  |  |  |  |  |  | 2 |
|  | United Kingdom |  |  |  | 6 |  |  |  |  |  |  | 6 |
| Oceania | Australia |  |  | 1 |  |  |  |  |  |  |  | 1 |
| Total |  | 16 | 24 | 269 | 385 | 246 | 160 | 234 | 135 | 50 | 2 | 1521 |

**Table** **S7.** Characteristics of three recombination forms in eight CVA6 sequences.

| **Recombinant Forms** | **Strain** | **Breakpoint Positions^a^** | **Covering**  **Regions^b^** | **Recombinant Donors (Similarity)** | **Methods^c^** | | | | | | |
| --- | --- | --- | --- | --- | --- | --- | --- | --- | --- | --- | --- |
|  |  |  |  |  | **RDP** | **GENECONV** | **BootScan** | **MaxChi** | **Chimaera** | **Siscan** | **3Seq** |
| RF-Z | CVA6/C-Tan/BJ/201907 | 5926–7360  (5925–7361) | 3C, 3D,  3' UTR | Major: CVA6_OR394973 (97.1%)  Minor: CVA4_MN964078 (96.8%) | 2.169×10^-111^ | 1.057×10^-85^ | 3.117×10^-108^ | 1.000×10^-23^ | 3.839×10^-25^ | 1.947×10^-26^ | 4.449×10^-8^ |
| RF-AA | CVA6/C-Tan/BJ/202308 | 6156–7321  (6207–7374) | 3D,  3' UTR | Major: CVA6_OR734735 (97.7%)  Minor: Unknown | 5.743×10^-4^ | NA | 1.036×10^-4^ | 2.132×10^-11^ | 1.259×10^-3^ | 5.010×10^-24^ | 2.545×10^-30^ |
| RF-AB | CVA6/C-Tan/BJ/202301 | 5760–7389  (5777–7408) | 3C, 3D,  3' UTR | Major: CVA6_OL830027 (97.6%)  Minor: CVA4_ON730851 (96.7%) | 4.988×10^-116^ | 8.529×10^-86^ | 1.069×10^-112^ | 1.739×10^-25^ | 1.606×10^-27^ | 6.046×10^-25^ | 3.846×10^-8^ |
| RF-AB | CVA6/C-Tan/BJ/202302 | 5763–7384  (5777–7400) | 3C, 3D,  3' UTR | Major: CVA6_OL839945 (97.9%)  Minor: CVA4_MN964080 (96.7%) | 4.092×10^-116^ | 2.222×10^-87^ | 2.380×10^-112^ | 8.492×10^-28^ | 2.076×10^-27^ | 3.213×10^-27^ | 4.051×10^-8^ |
| RF-AB | CVA6/C-Tan/BJ/202309 | 5767–7413  (5781–7429) | 3C, 3D,  3' UTR | Major: CVA6_OL830031 (97.2%)  Minor: CVA4_ON730851 (96.8%) | 9.297×10^-113^ | 1.260×10^-84^ | 7.381×10^-106^ | 6.073×10^-27^ | 6.934×10^-28^ | 1.483×10^-27^ | 4.386×10^-8^ |
| RF-AB | CVA6/C-Tan/BJ/202310 | 5621–7197  (5781–7359) | 3C, 3D,  3' UTR | Major: CVA6_OL839937 (97.6%)  Minor: CVA4_MN964080 (96.9%) | 9.032×10^-115^ | 9.409×10^-87^ | 1.178×10^-97^ | 6.917×10^-27^ | 7.360×10^-27^ | 3.467×10^-27^ | 2.100×10^-8^ |
| RF-AB | CVA6/C-Tan/BJ/202312 | 5454–7107  (5777–7431) | 3C, 3D,  3' UTR | Major: CVA6_MZ491032 (97.5%)  Minor: CVA4_MN964080 (96.7%) | 3.531×10^-114^ | 5.211×10^-82^ | 2.193×10^-75^ | 3.411×10^-28^ | 3.335×10^-28^ | 4.272×10^-29^ | 4.309×10^-8^ |
| RF-AB | CVA6/C-Tan/BJ/202316 | 5781–7421  (5777–7419) | 3C, 3D,  3' UTR | Major: CVA6_MN845811 (97.5%)  Minor: CVA4_ON730851 (97.2%) | 3.120×10^-118^ | 2.556×10^-93^ | 7.157×10^-109^ | 6.671×10^-27^ | 7.234×10^-28^ | 3.422×10^-28^ | 4.324×10^-8^ |

^a^ represent the breakpoint position without gaps (the numbers within brackets represent the breakpoint position aligned with AY421764);

^b^ represent the genomic structure of enterovirus which was related to recombination;

^c^ represent the P-value given by RDP4 packages.

**Table S8.** Characteristics of amino acid mutations in recombinant and non-recombinant regions for the RF-Z and RF-AB sequences.

| **Strain name** | **Recombinant region** | | **Non-recombinant region** | | ***χ*² Value** | ***P* Value** |
| --- | --- | --- | --- | --- | --- | --- |
|  | Mutations**^a^** | No changes^b^ | Mutations | No changes |  |  |
| CVA6/C-Tan/BJ/201907 | 21 | 454 | 23 | 1,703 | 18.136 | < 0.001 |
| CVA6/C-Tan/BJ/202301 | 23 | 501 | 22 | 1,655 | 18.88 | < 0.001 |
| CVA6/C-Tan/BJ/202302 | 22 | 502 | 22 | 1,655 | 16.981 | < 0.001 |
| CVA6/C-Tan/BJ/202309 | 22 | 501 | 23 | 1,655 | 16.011 | < 0.001 |
| CVA6/C-Tan/BJ/202310 | 21 | 502 | 22 | 1,656 | 15.222 | < 0.001 |
| CVA6/C-Tan/BJ/202312 | 22 | 502 | 21 | 1,656 | 18.093 | < 0.001 |
| CVA6/C-Tan/BJ/202316 | 22 | 502 | 23 | 1,654 | 15.932 | < 0.001 |

^a^ Number of amino acid mutations compared to KM114057 (the earliest reference strain of the sub-genotype D3).

^b^ Number of sites without amino acid mutations compared to KM114057.
